# A Comparative Study between Beeswax and Glycerol Monostearate for Food-Grade Oleogels

**DOI:** 10.3390/gels10040214

**Published:** 2024-03-22

**Authors:** Francesca Malvano, Donatella Albanese, Luciano Cinquanta, Sara Liparoti, Francesco Marra

**Affiliations:** 1 Department of Industrial Engineering, University of Salerno, 84084 Fisciano, Italy; fmalvano@unisa.it (F.M.); sliparoti@unisa.it (S.L.); fmarra@unisa.it (F.M.); 2 Department of Agricultural, Food and Forest Sciences, University of Palermo, 90121 Palermo, Italy; luciano.cinquanta@unipa.it

**Keywords:** beeswax, glycerol monostearate, oleogel, olive oil

## Abstract

With the aim to produce solid fats with a high percentage of unsaturated fatty acids, oleogels based on olive and peanut oil with different concentrations of beeswax (BW) and glycerol monostearate (GMS) as oleogelators were studied and compared. The critical oleogelator concentration for both BW and GMS was 3%. Thermal properties of the developed GMS-based oleogels pointed to a polymorphic structure, confirmed by the presence of two exothermic and endothermic peaks. All developed oleogels released less than 4% of oil, highlighting their high oil binding capacity. A morphology evaluation of oleogels showed platelet-like crystals, characterized by a cross-sectional length of 50 μm in BW-based oleogels and irregular clusters of needle-like crystals with a higher diameter in GMS-based oleogels. BW-based oleogels showed a solid fat content ranging from 1.16% to 2.27%, and no solid fat content was found at 37 °C. GMS-based oleogels reached slightly higher values of SFC that ranged from 1.58% to 2.97% at 25 °C and from 1.00% to 1.75% at 37 °C. Olive oil-based oleogels with GMS showed higher firmness compared with BW-based ones. The stronger structure network in olive oil/GMS-based oleogels provided a real physical barrier to oxidants, showing a high oxidation stability.

## 1. Introduction

Fats and edible oils are essential nutrients in the human diet, even though a diet with an abundant amount of saturated fatty acids has been associated with diabetes, obesity, cancer, and cardiovascular diseases [1,2]. For these reasons, the World Health Organization recommends that saturated fatty acid (SFA) consumption should be less than 10% of total energy consumption. However, the formulation of most lipid-based food products requires the use of semi-solid fats with a high percentage of SFA due to their important role in the sensory and technological quality of food products [3]. 

So, the direct replacement of saturated fats with unsaturated ones could bring technological problems, including adverse changes to mechanical and rheological properties, other than modifying the thermal properties of the fat substances with a high impact on the flavor substances released during chewing [4,5].

An emerging class of structured lipids called “oleogels” seems to offer an interesting way to structure liquid oil as a solid-like material. Oleogels can be defined as semi-solid systems characterized by a continuous phase of vegetable liquid oil where a three-dimensional network (composed of an oleogelator) is responsible for the physical entrapment of the liquid [6,7].

The normal phase separation into aggregated gelator molecules and liquid solvent phase is avoided in oleogels due to the organization of aggregated gelator molecules into an interconnected solid-like three-dimensional (3D) network, resulting in the formation of strong or weak gels depending on the gelator–gelator interactions and the solvent properties. In this regard, the fatty acid composition of oils as the main distinguishing feature of all edible oils is considered one of the most important factors influencing oleogel properties. Higher unsaturation degrees induce higher conformational freedom, leading to a more crooked spatial arrangement, decreasing the interaction energy, and giving a more hydrophobic behavior of the oil [8]. The increasing hydrophobicity facilitates the formation of a greater number of junction zones and therefore will produce stronger gels [7]. It is worth noting that the non-lipid polar materials, present in low amounts in the oils, can have their role.

Oleogels can be prepared by different types of structurants (oleogelators), which are responsible for different gelation mechanisms. The most well-known gelation mechanisms include fatty acid crystallization, self-assembled fibrillar networks, polymeric networks, and reverse spherical micelles [6]. The ability of a structuring agent to form a gel in a liquid medium has been attributed to its capability to interact both with the continuous phase and with itself, leading to network formation. Commonly, very low amounts of oleogelator ranging from 5 to 15% for natural gums, 7 to 13% for polysaccharides, and 5 to 10% for protein-based oleogelators are able to entrap liquid oil and create a crystalline three-dimensional network [9]. 

In the related literature, many studies have been conducted regarding the role of natural waxes and monoglycerides of fatty acid able to structure vegetable oils, with promising findings for food applications [9,10,11,12,13,14,15,16,17,18,19,20,21,22,23,24]. 

The gelation mechanism of these components when mixed with oil requires the melting of the waxes and monoglycerides upon heating and their precipitation during cooling to form solid nuclei. The onset of crystal growth results in strong crystalline interactions and formation of supramolecular entities, which entrap liquid oil into a three-dimensional network. Strong primary sintered connections and weak secondary van der Waals bonds are the main driving forces for network formation [10]. 

Beeswax (BW) is a common edible gelator for different edible oils. BW-based oleogels involving camelia oil, soybean oil, sunflower oil, flaxseed oil, and a mixture of fish, olive, and linseed oils have been studied by different authors, demonstrating good oil binding ability, texture, thermal behavior, and stability [11,12,13,14].

Several studies have also reported the use of monoglycerides as gelators in oleogels. Ferro et al. [15] dealt with the role of sunflower oil, high oleic sunflower oil, and coconut oil on the formation of the GMS gel network, highlighting that the long- and medium-chain fatty acids of coconut oil resulted in a gel with poor stability, in contrast with high oleic sunflower oil, which favored the packaging of GMS molecules and promoted the formation of a structured system. Previous studies [16,17] showed that the increment in the cooling rate for the formation of high oleic sunflower oil-based oleogel allowed for improving the network structure, with an increment in oil binding capacity, hardness, and elasticity. Recent studies focus on the study of oleogels not only characterized by good stability but the ability to bring benefits to human health.

Olive oil, thanks to its well-known positive health effects associated with its main (oleic, linoleic, and linolenic acids) and minor (oleuropein, phytosterol, tocopherol, carotenoid, chlorophyll, and aromatic) components [25,26], and peanut oil, characterized by a high amount of oleic and linoleic acids [27], could be interesting candidates for promoting the nutritional benefits of oleogels [28]. In the literature, different studies focus on the development of oleogels using olive oil as a solvent [16,18,29,30]. To our knowledge, there is a gap in the knowledge regarding oleogels made with peanut oil. Moreover, information is missing regarding the influence of different concentrations of BW and GMS on the physicochemical and thermal properties of oleogels in peanut oil and olive oil.

For this reason, the present study aimed to contribute to the data regarding common physicochemical and thermal features (oil binding capacity, oxidative stability, firmness, colour, crystal morphology, and thermal behavior) of such oleogels, thus providing practical information for their possible applications in the food sector and suggesting criteria for the optimization of important factors influencing the quality parameters and shelf life of fat-based products such as creams, chocolate, and sweet bakery products.

## 2. Results and Discussion

### 2.1. Fatty Acid Composition

The fatty acid profiles of olive and peanut oils are presented in Table 1.

As expected, for both samples, MUFA represented the main group of fatty acids, with oleic acid (C18:1) being predominant (73.90% and 68.30% for olive and peanut oil, respectively), followed by PUFA, of which linoleic acid (C18:2) was the most abundant.

Both samples presented a total saturated fatty acid content of less than one-sixth of the total fatty acid content (14.00% and 12.20% for olive and peanut oil, respectively). Among SFA, the primary acid was palmitic acid (16:0), followed by stearic acid (18:0). Only traces of gamma-linolenic acid (C18:3) and cis-eicosenoic acid (C20:1) were found.

Peanut oil showed a higher MUFA/SFA ratio than olive oil. The MUFA/SFA ratio was characteristic of each oil being an important nutritional parameter because it is associated with a reduced risk of all-cause mortality [31].

### 2.2. Physicochemical and Structural Properties of Beeswax and Glycerol Monostearate Oleogels

**Figure 1:**
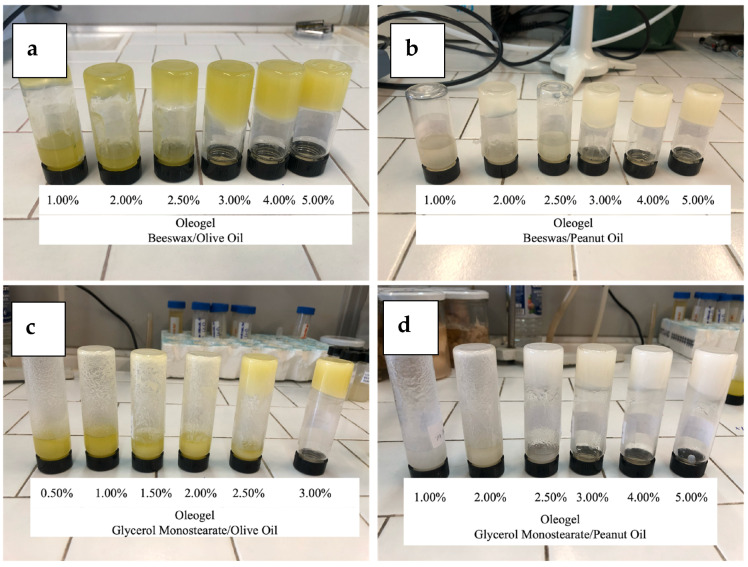
Oleogels prepared in this study: (**a**) beeswax/olive oil; (**b**) beeswax/peanut oil; (**c**) glycerol monostearate/olive oil; (**d**) glycerol monostearate/peanut oil.

The oleogel structure formation depends on both the structure of the oleogelator and the concentration in the medium. As shown in Figure 1, for both OO and PO, the minimum concentration of oleogelators was 3.00%. As reported in the literature, BW and GMS are used as efficient oleogelators in different oils, such as sunflower oil, coconut oil, and medium- and long-chain triglycerides, at concentrations lower than 5.00% [15,16,17,18,19,20,21,22,23,24,25,26,27,28,29,30,31,32].

The oleogel crystalline network is responsible for the physical entrapment and immobilization of the organic solvent. Oil binding capacity is an indicator of the stability of the oleogel structure; it indicates the capability of a three-dimensional gel structure to keep the liquid phase. To evaluate the stability of the oleogel structure in the entrapment of the oil phase, oil binding capacity was measured on fresh oleogel samples and after 6 months of storage at room temperature.

All fresh (t = 0) oleogels showed high OBC values, since the oil released after centrifugation was lower than 4.00%. Regarding the stability over time, no significant differences (*p* < 0.05) were observed after 6 months of storage (Table 2). These results confirm the high capability of developed oleogels to keep oil and their high physical stability.

As reported by Blake and Marangoni [33], the oil binding capacity of an oleogel is correlated with a total crystalline surface area with homogeneously distributed small crystals. In particular, the capability of GMS to gel vegetable oils is associated with the formation of inverse lamellar phases stabilized by strong hydrogen bonds [34], while the crystallization phenomena of BW in liquid oils generally result in gels where the network based on the van der Walls interaction between crystals and crystalline aggregate immobilizes the liquid oil into a tridimensional structure [35].

Regarding the influence of the concentration of oleogelators on the capability of the oleogels to entrap the oil, no significant differences (*p* < 0.05) were found among the oleogels made with the same structuring agent, suggesting that 3.00% was the suitable concentration able to develop a self-supporting network.

**Figure 2:**
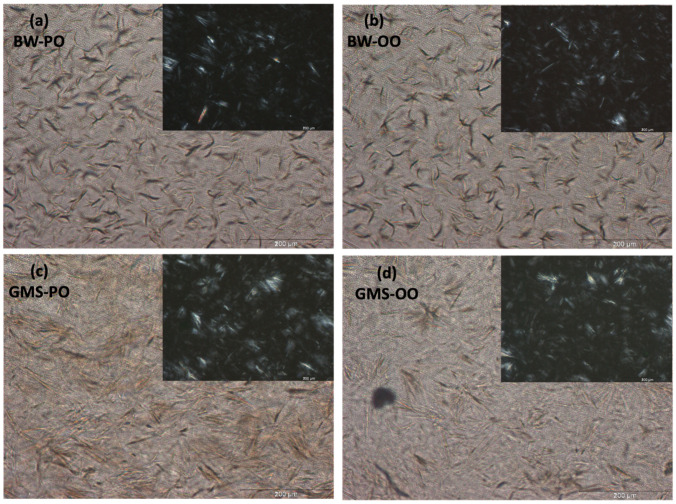
Bright field light microscopy of beeswax (5.00%)/peanut oil (**a**); beeswax (5.00%)/olive oil (**b**); glycerol monostearate (5.00%)/peanut oil (**c**); glycerol monostearate (5.00%)/olive oil (**d**). Inserts: same images using polarizing lenses.

The above was confirmed by a crystal morphology evaluation of developed oleogels observed through the microscope. Figure 2 shows the crystalline microstructure of the BW- and GMS-based oleogels at the same concentration of oleogelators (5.00%), highlighting how the oleogelator type affected the morphology of the crystals.

Platelet-like crystals, characterized by a cross-sectional length of approximately 50 μm, were observed in BW-based oleogels. Our results are in agreement with previous authors [30,33], who observed platelet-like crystals in beeswax/olive oil oleogels with dimensions ranging from 30 to 50 μm. Contrarily, Papadaki et al. [19] observed flaked-like crystals in oleogels made with bio-based wax esters, while Pang et al. [13] observed needle-like crystals in beeswax oleogel with different types of vegetable oil, including camellia oil, soybean oil, sunflower oil, and flaxseed oil. The different morphologies observed by the authors could be due, as suggested by Blake and Marangoni [23], to the technological limits of the traditional optical microscopy techniques regarding the identification of the true morphology of the crystals. Irregular clusters of needle-like crystals with mean diameters higher than 50 μm were formed in GMS-based oleogel; this kind of structure was previously reported for GMS-based oleogels with different solvents such as olive and corn oil [21], sunflower oil [36], canola oil, and soybean oil [34], in which firmer and stronger gel networks were highlighted.

The different crystal morphology observed for BW- and GMS-based oleogels can be due to the different compositions of oleogelators. The main components of BW are fatty hydrocarbons, free fatty acids, free fatty alcohols, fatty acids moieties, and fatty alcohol moieties [13,37], in contrast with GMS, which is a monoglyceride produced from an esterification reaction between glycerol and stearic acid [15].

Regarding the influence of oil composition on the oleogel crystal microstructure, Ferro et al. [15] observed that sunflower and high oleic sunflower oils, rich in long-chain monounsaturated fatty acids, favored the packing of GM crystals in a very cohesive gel, promoting the formation of a well-structured system, in contrast with coconut oil (COO) rich in medium-chain saturated fatty acid, which was unable to form a stable gel. A comparison between medium-chain triglyceride (MCT) and long-chain triglyceride (LCT)-based oleogels highlighted the capability of LCT oleogel to create a strong network [32]. Regarding the fatty acid composition, no differences were found in the crystal morphology influence on oleogels based on vegetable oils with a different unsaturation degree such as sunflower and camelia oils [13]. However, our micrographs did not show any differences between OO and PO for both BW and GMS, probably due to the similar unsaturation degree and length of fatty acid chain.

The evaluation of oleogel color highlighted how the color of the oil totally affected the color of the oleogel (Table 2). The results, in fact, did not show any significant difference in the values of L, a*, and b* among olive oil and all the olive oil-based oleogels, or among the peanut oil and the oleogels prepared with it. Regardless of the type of oleogelator used, the olive oil-based oleogels appeared with yellowish colors, while the peanut oil-based oleogels showed a creamy-white color, thus avoiding any difficulty in food applications.

### 2.3. Thermal Properties Evaluation

Crystallization and melting curves for BW, GMS, and oleogels are shown in the supplementary data (Figures S1 and S2). Crystallization temperatures (Tc), melting temperatures (Tm), and enthalpies related to each thermal process for neat BW and GMS and for all developed oleogels are reported in Table 3.

According to Gomez-Estaca [11] and Yilmaz and Ogutcu [30], neat BW showed the presence of a single crystallization and melting transition close to 58.28 °C and 63.25 °C, respectively. On the contrary, other authors [13,32,38] found two different peaks for both crystallization and melting transition. The different thermal behavior of neat BW could be due to the different composition in fatty acid esters, alcohols, free wax acids, and n-alkanes with different dropping point, acid, ester, and saponification values [39].

On the contrary, neat GMS presented two well-defined melting peaks in the heating step at 16.68 °C and 63.15 °C and two crystallization peaks in the cooling step at 62.27 °C and 17.23 °C. The presence of two defined peaks during the cooling step of GMS was observed by Lupi et al. [40] and Ferro et al. [15]. Based on other literature studies, the first exothermic peak corresponded to the crystallization of the aliphatic tails of the mixed lamellar structure and the second peak was associated with the polymorphic transition into the sub-α structure [34,41].

BW-based oleogels showed significantly (*p* < 0.05) lower temperatures of crystallization and melting transitions than those obtained for pure BW, probably due to the dilution effect. As shown in Table 3, olive oil-based oleogels exhibited crystallization temperatures ranging from 16.94 °C for 3.00% BW to 20.71 °C for 5.00% BW, while melting temperatures ranged from 31.37 °C for 3.00% BW to 36.42 °C for 5.00% BW. Peanut oil-based oleogels showed crystallization and melting temperatures close to those obtained for olive oil-based oleogels. In particular, crystallization temperatures ranged from 20.33 °C for 3.00% BW to 23.36 °C for 5.00% BW, while melting temperatures ranged from 35.10 °C for 3.00% BW to 37.11 °C for 5.00% BW. In all cases, both the crystallization and melting temperatures of the BW-based oleogels moved toward a high temperature with the increase in beeswax concentration, according to Yilmaz and Ogutcu [30], Yilmaz and Ogutcu [42], and Martins et al. [32]. Doan et al. [43] attributed this behavior to the dilution of the bulk wax in vegetable oil; as the concentration of beeswax increased, ΔH of the oleogels increased.

A different thermal behavior was observed for GMS-based oleogels (Table 3), with two endothermic peaks observed in the second heating probably due to the polymorphic nature of pure GMS that involved the presence of two exothermic and endothermic peaks in the oleogels. As for BW-based oleogels, also in this case, temperatures of crystallization and melting of GMS-based oleogels significantly (*p* < 0.05) lower than those obtained for pure GMS were observed; moreover, these temperatures increased with the increase in oleogelator concentration.

The crystallization process in different steps in GMS-based oleogels could be due to the high degree of crystalline disorder present in the structure, previously highlighted in polarized light microphotographs. Firstly, a group of GMS crystals started to form as the onset temperature was reached (close to 50 °C), regardless of the liquid phase used for the realization of oleogels. Then, another crystallization peak appeared close to 10 °C, supporting—for the investigated condition—the structuring process of both GMS-OO- and GMS-PO-based oleogels.

The melting temperatures in GMS-based oleogels ranged from 47.29 °C to 50.10 °C for olive oil-based oleogels and from 44.58 °C to 48.52 °C for peanut oil-based oleogels. BW-oleogel crystallization and melting peaks increased with the amount of oleogelators.

Regarding the influence of oil composition on the oleogel thermal properties, BW crystals in oleogel systems with higher polyunsaturated degrees had higher melting and crystallization temperatures [37]. However, as found in this study, no differences in thermal properties were observed for oleogels based on vegetable oils similar in fatty acid composition [15,37]. It is worth noting that different studies [12,13,32] have reported very similar thermograms of BW-based oleogels formulated with different oils characterized by similar fatty acid compositions, indicating that the thermal curve largely depends on the oleogelator regardless of the oil phase.

Enthalpy value in the melting peak observed in GMS oleogels pointed to higher values than those detected for BW-based ones, indicating a different capability of two oleogelators to produce a stronger crystalline network.

This behavior greatly affected the solid fat content (SFC) of the oleogels (another important thermal property of a fat that defines the percentage of the solid parts of fats at a certain temperature).

**Figure 3:**
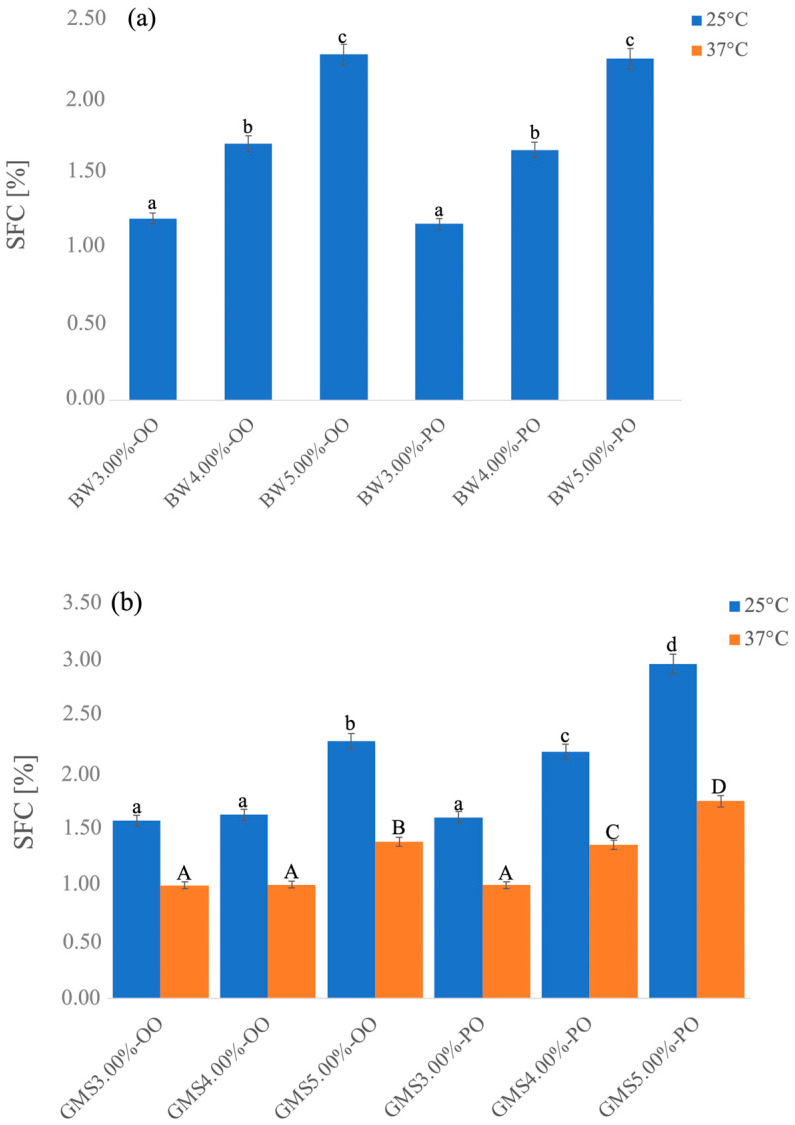
SFC of BW (**a**) and GMS (**b**)-based oleogels evaluated at 25 °C and 37 °C. Different letters (a–d) reveal significant differences (*p* < 0.05) among the samples at 25 °C; different letters (A–D) reveal significant differences (*p* < 0.05) among the samples at 37 °C.

The SFC of all types of oleogels was found to increase with the increase in oleogelator content (Figure 3). In particular, SFC at 25 °C of BW-based oleogels ranged from 1.16% to 2.27%, and no SFC was found at 37 °C (Figure 3a). On the other hand, GMS-based oleogels reached slightly higher values of SFC, ranging from 1.58% to 2.97% at 25 °C and from 1.00% to 1.7 5% at 37°C (Figure 3b).

SFC at different temperatures is an important parameter for qualifying oleogels for specific food preparation. At mouth temperature (37 °C), most fats should melt in order to ensure the flavor release [44].

The higher content of SFC in GMS-based oleogels should favor their application in substitution of high melting fats such as shortenings for puff pastry products. In contrast, BW-based oleogels should find a better use for the development of cocoa-based creams, for which the low SFC at room and mouth temperatures affects the spreadability and the release of fat-soluble flavor compounds [45].

### 2.4. Peroxide Value Evaluation

The oxidation stability of fresh oils and oleogels was evaluated after one month of storage at 25 °C by peroxide value (PV), which is an important parameter for the evaluation of the amount of primary products produced during lipid oxidation.

**Figure 4:**
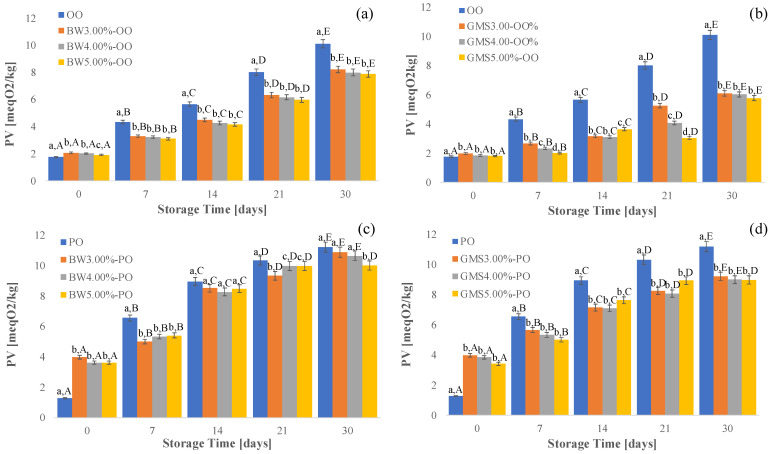
Changes in PV during 1 month of storage of BW-based oleogels with olive oil (**a**), GMS-based oleogels with olive oil (**b**), BW-based oleogels with peanut oil (**c**), GMS-based oleogels with peanut oil (**d**). Different letters (a–d) reveal significant differences (*p* < 0.05) among the samples for each storage time; different letters (A–E) reveal significant differences (*p* < 0.05) among treatments during the storage time.

The results (Figure 4) highlight that, on the first day of the storage period, all oleogels exhibited higher peroxide values than the fresh oil. It could be explained by the fact that, during the oleogel preparation, the oils were exposed to a high temperature for a fixed time [46].

However, as expected, the PV of all samples gradually increased with increasing storage time. It is worth noting that the peroxide value of PO and OO increased at a faster rate during storage compared with the respective oleogels. This behavior could be due to the structuring of the oils allowing the reduction in exposure of the fat liquid phase to oxygen, thus slowing down the oxidative phenomena.

In particular, during the storage period, at the same percentage as the oleogelator, PO-based oleogels showed higher PV values than OO-based oleogels due to a higher unsaturation degree than olive oil.

The best result was obtained for GMS (5.00%)-based oleogel with olive oil, in which the lowest (*p* < 0.05) increase in peroxide value during one month of storage was recorded. Therefore, the three-dimensional network formed by GMS-based oleogels was able to provide a real physical barrier to protect the organic solvent from light and oxygen effects during storage.

### 2.5. Oleogel Firmness

Textural properties of organogels are quite important factors for understanding their influence on the quality parameters of some fat-based products (creams, chocolate, and ice cream) [47].

**Figure 5:**
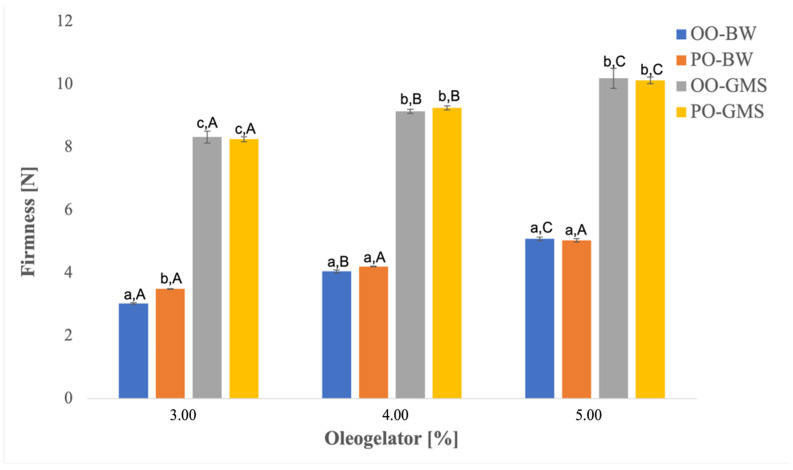
Firmness of oleogels prepared with different concentrations of oleogelators. Different letters (a–c) reveal significant differences (*p* < 0.05) among the samples for each oleogelator %; different letters (A–C) reveal significant differences (*p* < 0.05) among samples at different oleogelator %.

The firmness results of all developed oleogels are reported in Figure 5. As expected, the oleogel firmness increased (*p* < 0.05) with the increase in oleogelator concentration for both BW and GMS-based oleogels. Also, the oleogels made with GMS showed higher firmness than BW-based ones, while the type of oil did not affect this parameter. The higher firmness of GMS-based oleogels could also explain their higher oil binding capacity and higher melting temperature. It has been studied that the firmness of an oleogel is strongly influenced by some conditions, such as structuring composition and crystallinity degree [48].

Moreover, the comparison between the firmness and PV values at 21 days of storage in all prepared oleogels pointed to the higher the firmness the lower the oxidation state of the sample, suggesting that a firmer structure of oleogels is able to provide an effective physical barrier to light and oxygen responsible for oxidation phenomena in the lipid phase.

## 3. Conclusions

The critical concentrations, thermal properties, structural characteristics, and oxidative stability of oleogels prepared with olive oil and peanut oil using beeswax and glycerol monostearate as oleogelators were investigated in this study. Both oils showed a solid-like material structure when the oleogelator concentration was higher than 3.00%.

The thermal evaluation highlighted the mixed lamellar structure of GMS, resulting in the presence of two crystallization and two melting temperatures in the oleogels. Moreover, GMS-based oleogels, thanks to their well-structured crystalline network, showed higher oil binding capacity, higher solid fat content at 25 °C and 37 °C, and higher melting and crystallization points with respect to BW-based oleogels.

Enthalpy values in the melting peak observed in GMS-based oleogels pointed to higher values than those detected for BW-based ones, indicating the different capabilities of two oleogelators to produce a stronger crystalline network.

Moreover, the higher firmness of GMS-based oleogels resulted in lower peroxide values, suggesting that the three-dimensional network formed by GMS is able to provide a real physical barrier to protect the organic solvent from light and oxygen effects during storage, slowing down oxidation phenomena.

Finally, the evaluation of oleogel color highlighted that the color of the oil totally affected the color of the oleogels. Regardless of the type of oleogelator used, the olive oil-based oleogels appeared with yellowish colors, while the peanut oil-based oleogels showed a creamy-white color, thus avoiding any difficulty in food applications. Regarding the influence of OO and PO on the oleogel properties, the obtained results suggest that both oils are suitable solvents for oleogel manufacturing with GMS and BW concentrations of at least 3.00%. These findings indicate that PO and OO oleogels with suitable oil binding capacity and stability in oxidation and firmness could represent fat-based alternatives in the production of food products that require the use of solid fat to reach specific physical properties that affect their quality, such as spreads, chocolate fillings, and sweet bakery products.

## 4. Materials and Methods

### 4.1. Materials

Beeswax (dropping point 63 °C, acid value 17 mg KOH per g, ester value 73.4 mg KOH per g, and saponification value 90.4 mg KOH per g) was purchased from ACEF (Piacenza, Italy) and pure glycerol monostearate (pure power with 99.99% total monoglycerides) was obtained from Axenic Health Solutions (Plano, TX, USA). Commercial olive oil and peanut oil were obtained from a local market.

### 4.2. Fatty Acid Composition Analysis

The fatty acid composition of the olive oil and peanut oil was determined by analyzing the fatty acid methyl esters (FAMEs) obtained after transesterification, according to Memoli et al. [49].

The fatty acid composition was analyzed using an Agilent 6890 N gas chromatograph (Agilent Technologies, Milan, Italy), equipped with a capillary column (60 m × 0.25 mm i.d., 0.25 μm film thickness fused silica capillary column Supelcowax^®^ 10 (Supelco, Bellefonte, PA, USA)) and a flame ionization detector (FID) and split/spitless injector. The temperature was increased from 160 °C to 220 °C at 1 °C/min until reaching the final temperature of 230 °C at 2 °C/min for 28 min. The split ratio was 20:1, and the injected volume was 1 μL. FAMEs, dissolved in hexane, were injected (1 μL) in a split injection mode at a split ratio of 20:1. Detector and injector temperature was 250 °C and helium was used as the carrier gas at 1 ml/min. Identification was conducted by comparing retention times with standards of different fatty acids. Results were expressed as g_fatty acid_/100 g_oil_.

### 4.3. Oleogel Preparation

Different concentrations of BW and GMS ranging from 0.50% to 5.00% (*w*/*w*) were dissolved in olive oil (OO) and peanut oil (PO) at 85 °C under magnetic stirring at 100 rpm. After the complete oleogelator dissolution, the mixtures were placed in a refrigerated bath at 5 °C for 30 min until the samples reached room temperature (25 ± 2 °C), recording an average cooling rate of 2 °C/min. The prepared oleogels were stored at room temperature for at least 24 h before being analyzed. The proper amount of structuring agent was defined from tilting tests to visually evaluate the minimal oleogelator amount (C*) able to produce oleogel. Twenty-four hours after the preparation, the tubes containing oleogel were inverted at 180° and a possible flow of sample was observed. The samples without flow were selected for the detection of C*. For each oleogelator (BW and GMS) and each oil (OO and PO), the following concentrations (3.00, 4.00, and 5.00%) were selected.

### 4.4. Oleogel Characterization

#### 4.4.1. Oil Binding Capacity

The oil binding capacity (*OBC*) of oleogels was evaluated following the procedure of Uslu and Ylmaz [50] with some modifications. Amounts of 1 ml of melted oleogel (90 °C for 30 min) were placed into tared Eppendorf tubes (a). The tubes were stored at room temperature overnight for complete gelation. After that, the tubes were weighed (b) and centrifuged (10,000 rpm for 15 min) before drainage of the released liquid oil on the paper cloth. The tubes were weighed (c) again and the *OBC* was calculated according to the following equation:

(1) $$OBC\;(\%)=100-\left[\left[\frac{\left(b-a\right)-\left(c-a\right)}{b-a}\right]\times 100\right]$$

#### 4.4.2. Light Microscopy

The crystal morphology of oleogels was studied using a light microscope (Olympus BX-41, Olympus Corporation, Waltham, MA, USA) in bright field and polarized modes, connected to a CCD camera, which allowed taking photographs of the sample under test. The melted oleogels were poured onto the glass slides and stored at 5 °C for 24 h before being analyzed. The crystals were photographed at 100 times magnification at room temperature.

#### 4.4.3. Color Evaluation

The color of the oleogels was evaluated with a Minolta CR-400 colorimeter (Konica Minolta Sensing, Osaka, Japan) according to CIE standards and L, a*, and b* parameters were registered on different points on the samples at room temperature.

#### 4.4.4. Thermal Analysis

The thermal behavior of oleogels was analyzed using differential scanning calorimetry (Q Series DSC, TA Instruments, New Castle, Delaware, USA). A total of 3–5 mg of sample was loaded in an aluminum pan and nitrogen was used at a flow rate of 50 mL/min. An empty pan was used as reference. Samples were initially equilibrated at 20 °C, heated to 80 °C at 10 °C/min and cooled to −20 °C at 10 °C/min, kept isothermally at −20 °C for 3 min, and reheated to 100 °C at 5 °C/min. Thermograms were analyzed by TA Universal Analysis 2000 (TA Instruments, New Castle, DA, USA) software, defining some properties from thermal curves as the crystallization temperature (Tc) associated with the heat flow of exothermic event and the melting peak temperature (Tm) associated with the heat flow of the endothermic event.

TA Universal Analysis software was also used for the evaluation of solid fat content by investigating the integral curves for melting, according to the following equation [35,36]

(2) $$SFC=100\;\frac{\int_{T}^{T_{f}}HdT}{\int_{T_{0}}^{T_{f}}HdT}$$

where *H* is the melting enthalpy and *T*_0_ and *T_f_* are the initial and final melting temperatures, respectively.

#### 4.4.5. Peroxide Value Evaluation

The oxidative stability of oils and oleogels was evaluated through the detection of the peroxide value (*PV*) at 0, 7, 15, and 30 days of storage in a dark place at 25 °C, according to AOAC (1999). An aliquot of 7 g of sample oil or oleogel was weighed. Then, 25 mL of acetic acid/chloroform solution (3:2, *v*/*v*) and 0.5 mL of saturated potassium iodide solution were added to the sample. The flask was closed and stirred for 1 min in the dark. Afterwards, 75 mL of distilled water and indicator starch solution was incorporated into the mixture. Finally, the sample was titrated with 0.01 N sodium thiosulfate until the color disappeared. The *PV* value was calculated according to the following equation:

(3) $$PV\;(\frac{meqO_{2}}{kg})=\frac{V\;\;N\;\;100}{A}$$

where *V* is the volume in mL of 0.01 N sodium thiosulfate solution, *N* is its normality, and *A* is the amount of the sample (g).

#### 4.4.6. Firmness Evaluation

The firmness of the oleogels was determined at room temperature by a texture analyzer (LRX Plus, Lloyd Instruments, Chicago, IL, USA). An amount of 25 g of melted oleogel sample was poured into a cylindrical-shaped container (35 mm in diameter and 50 mm high) and cooled as described above. The container was placed on the platform of the instrument equipped with a cylindrical probe (12 mm diameter), which was lowered 18 mm into the samples at a crosshead speed of 30 mm/min. Firmness was calculated by the maximum force, which was recorded from the graph of the force versus penetration distance [26].

### 4.5. Statistical Analysis

Experimental data were reported as mean and standard deviation calculated from three replicates. The one-way analysis of variance (ANOVA) and multiple comparisons (Duncan’s test) were carried out to evaluate whether differences among the samples were statistically significant (*p* < 0.05) by using JMP statistical software (v. 16.2) [51].

## Figures and Tables

**Table 1:** Fatty acid composition (%) of olive oil and peanut oil.

	Olive Oil [%]	Peanut Oil [%]
SFA	14.00 ± 0.01 ^a^	12.20 ± 0.01 ^b^
MUFA	75.30 ± 0.02 ^a^	68.40 ± 0.04 ^b^
PUFA	10.70 ± 0.01 ^a^	19.40 ± 0.02 ^b^
MUFA/SFA	5.38 ± 0.00 ^a^	5.61 ± 0.00 ^b^
Palmitic Acid C16:0	12.10 ± 0.01 ^a^	9.80 ± 0.01 ^b^
Palmitoleic Acid C16:1	1.40 ± 0.00 ^a^	0.10 ± 0.00 ^b^
Stearic Acid C18:0	1.90 ± 0.00 ^a^	2.40 ± 0.00 ^b^
Oleic Acid C18:1	73.90 ± 0.02 ^a^	68.30 ± 0.0 ^ba^
Linolenic Acid C18:3	-	1.20 ± 0.00
Linoleic Acid C18:2	9.90 ± 0.01 ^a^	17.30 ± 0.02 ^b^
Gamma-Linolenic Acid C18:3	0.80 ± 0.00 ^a^	0.20 ± 0.00 ^b^
Cis-Eicosenoic acid C20:1	-	0.70 ± 0.00

SFA—saturated fatty acids, MUFA—monounsaturated fatty acids; PUFA—polyunsaturated fatty acids. Different letters (a, b) in the same row mean statistical difference (*p* < 0.05) between the samples.

**Table 2:** Oil binding capacity (t = 0 and t = 6 months) and color parameters of developed oleogels.

Sample	Oil Binding Capacity [%] t = 0	Oil Binding Capacity [%] t = 6 Months	L	a	b
OO	nd	nd	50.15 ± 0.16 ^a^	−5.18 ± 0.14 ^a^	20.10 ± 0.05 ^a^
PO	nd	nd	64.06 ± 0.06 ^b^	−2.64 ± 0.28 ^b^	13.42 ± 0.17 ^b^
BW3.00%-OO	97.39 ± 0.95 ^aA^	96.12 ± 0.75 ^aA^	50.45 ± 1.23 ^a^	−5.12 ± 0.18 ^a^	19.85 ± 1.01 ^a^
BW4.00%-OO	97.00 ± 1.09 ^aA^	96.34 ± 0.78 ^aA^	50.22 ± 1.45 ^a^	−5.21 ± 0.11 ^a^	20.01 ± 1.02 ^a^
BW5.00%-OO	97.24 ± 1.06 ^aA^	97.01 ± 0.56 ^aA^	50.34 ± 1.32 ^a^	−5.11 ± 0.15 ^a^	20.11 ± 1.11 ^a^
BW3.00%-PO	97.16 ± 1.01 ^aA^	96.76 ± 1.34 ^aA^	64.12 ± 1.23 ^b^	−2.78 ± 0.12 ^b^	13.12 ± 1.07 ^b^
BW4.00%-PO	97.14 ± 0.93 ^aA^	96.56 ± 0.89 ^aA^	64.11 ± 1.45 ^b^	−2.81 ± 0.11 ^b^	13.89 ± 0.99 ^b^
BW5.00%-PO	97.06 ± 0.65 ^aA^	96.23 ± 0.78 ^aA^	64.03 ± 1.54 ^b^	−2.75 ± 0.13 ^b^	12.99 ± 1.05 ^b^
GMS3.00%-OO	99.85 ± 0.19 ^bB^	98.97 ± 0.34 ^bB^	50.32 ± 1.21 ^a^	−5.12 ± 0.11 ^a^	19.89 ± 1.23 ^a^
GMS4.00%-OO	99.47 ± 0.62 ^bB^	99.01 ± 0.56 ^bB^	50.01 ± 1.11 ^a^	−5.21 ± 0.09 ^a^	19.99 ± 1.25 ^a^
GMS5.00%-OO	99.93 ± 0.05 ^bB^	98.94 ± 0.42 ^bB^	50.89 ± 1.14 ^a^	−5.19 ± 0.12 ^a^	20.04 ± 1.31 ^a^
GMS3.00%-PO	98.48 ± 0.56 ^bB^	98.08 ± 0.56 ^bB^	64.12 ± 1.45 ^b^	−2.11 ± 0.11 ^b^	13.21 ± 1.02 ^b^
GMS4.00%-PO	98.52 ± 0.24 ^bB^	98.51 ± 0.65 ^bB^	64.19 ± 1.21 ^b^	−2.15 ± 0.09 ^b^	13.67 ± 1.04 ^b^
GMS5.00%-PO	98.93 ± 0.34 ^bB^	98.34 ± 0.55 ^bB^	64.78 ± 1.38 ^b^	−2.19 ± 0.11 ^b^	12.87 ± 1.08 ^b^

nd—not detected. Different letters (a, b) in the same column mean statistical differences (*p* < 0.05) among the samples. Different capital letters (A, B) in the same row mean statistical difference (*p* < 0.05) between the samples.

**Table 3:** Thermal properties of neat BW, BW-based oleogels, neat GMS, and GMS-based oleogels.

	Crystallization				
Sample	Tc_onset_ [°C]	Tc_1_ [°C]	Tc_2_ [°C]	ΔHc_1_ [J/g]	ΔHc_2_ [J/g]
BW	60.76 ± 0.45 ^a^	58.28 ± 0.23 ^a^	-	170.67 ± 5.67 ^a^	-
BW3.00%-OO	29.24 ± 0.12 ^b^	16.94 ± 0.12 ^b^	-	2.65 ± 0.01 ^b^	-
BW4.00%-OO	31.87 ± 0.31 ^c^	19.58 ± 0.23 ^c^	-	2.78 ± 0.03 ^c^	-
BW5.00%-OO	31.87 ± 0.40 ^c^	20.71 ± 0.21 ^d^	-	2.83 ± 0.02 ^d^	-
BW3.00%-PO	38.24 ± 0.23 ^e^	20.33 ± 0.15 ^e^	-	2.82 ± 0.01 ^e^	-
BW4.00%-PO	41.07 ± 0.41 ^f^	22.37 ± 0.36 ^f^	-	2.91 ± 0.05 ^f^	-
BW5.00%-PO	42.98 ± 0.09 ^g^	23.36 ± 0.39 ^g^	-	2.96 ± 0.03 ^g^	-
GMS	64.27 ± 0.56 ^a^	62.27 ± 0.56 ^a^	17.23 ± 0.56 ^a^	150.45 ± 4.56 ^a^	80.34 ± 12.56 ^a^
GMS3.00%-OO	46.12 ± 0.11 ^b^	41.99 ± 0.23 ^b^	10.03 ± 0.03 ^b^	2.82 ± 0.03 ^b^	1.13 ± 0.01 ^b^
GMS3.00%-OO	48.25 ± 0.10 ^c^	45.16 ± 0.12 ^c^	10.45 ± 0.02 ^c^	2.28 ± 0.02 ^c^	1.28 ± 0.02 ^c^
GMS5.00%-OO	48.95 ± 0.08 ^c^	45.42 ± 0.35 ^c^	11.33 ± 0.08 ^d^	2.60 ± 0.05 ^d^	1.45 ± 0.05 ^d^
GMS3.00%-PO	45.82 ± 0.15 ^d^	37.21 ± 0.56 ^d^	10.59 ± 0.05 ^e^	2.06 ± 0.01 ^e^	0.38 ± 0.01 ^e^
GMS4.00%-PO	43.29 ± 0.11 ^e^	39.72 ± 0.23 ^e^	11.55 ± 0.03 ^f^	2.56 ± 0.06 ^f^	0.57 ± 0.01 ^f^
GMS5.00%-PO	50.06 ± 0.09 ^f^	43.09 ± 0.45 ^f^	12.44 ± 0.01 ^g^	2.88 ± 0.02 ^g^	0.86 ± 0.02 ^g^
Melting					
	Tm_1onset_ [°C]	Tm_1_ [°C]	Tm_2_ [°C]	ΔHm_1_ [J/g]	ΔHm_2_ [J/g]
BW	51.23 ± 2.67 ^a^	63.25 ± 0.98 ^a^	-	160.23 ± 3.45 ^a^	-
BW3.00%-OO	20.22 ± 0.34 ^b^	31.37 ± 0.67 ^b^	-	2.81 ± 0.04 ^b^	-
BW4.00%-OO	20.46 ± 0.23 ^b^	35.01 ± 0.45 ^c^	-	2.82 ± 0.03 ^b^	-
BW5.00%-OO	20.17 ± 0.12 ^b^	36.42 ± 0.23 ^d^	-	2.80 ± 0.06 ^b^	-
BW3.00%-PO	20.06 ± 0.16 ^b^	35.10 ± 0.56 ^c^	-	2.80 ± 0.02 ^b^	-
BW4.00%-PO	20.10 ± 0.15 ^b^	36.44 ± 0.24 ^d^	-	2.80 ± 0.03 ^b^	-
BW5.00%-PO	20.79 ± 0.21 ^b^	37.11 ± 0.54 ^e^	-	2.83 ± 0.04 ^b^	-
GMS	10.23 ± 1.03 ^a^	16.68 ± 0.87 ^a^	63.15 ± 0.56 ^a^	81.67 ± 5.23 ^a^	150.45 ± 12.45 ^a^
GMS3.00%-OO	7.34 ± 0.18 ^b^	11.43 ± 0.05 ^b^	47.29 ± 0.13 ^b^	11.37 ± 0.06 ^b^	1.05 ± 0.01 ^b^
GMS4.00%-OO	6.84 ± 0.14 ^c^	12.75 ± 0.07 ^c^	48.90 ± 0.15 ^c^	8.34 ± 0.09 ^c^	1.24 ± 0.03 ^c^
GMS5.00%-OO	9.87 ± 0.19 ^d^	12.29 ± 0.10 ^d^	50.10 ± 0.20 ^d^	12.16 ± 0.11 ^d^	1.51 ± 0.02 ^d^
GMS3.00%-PO	5.27 ± 0.19 ^e^	7.21 ± 0.03 ^e^	44.58 ± 0.16 ^e^	7.49 ± 0.04 ^e^	1.55 ± 0.01 ^e^
GMS4.00%-PO	6.83 ± 0.18 ^f^	12.71 ± 0.04 ^c^	45.94 ± 0.12 ^f^	7.39 ± 0.03 ^f^	1.19 ± 0.04 ^f^
GMS5.00%-PO	5.22 ± 0.15 ^g^	13.76 ± 0.05 ^f^	48.52 ± 0.14 ^g^	9.14 ± 0.01 ^g^	1.10 ± 0.01 ^g^

Different letters (a–g) in the same column mean statistical differences (*p* < 0.05) among the samples.

## Data Availability

The datasets generated during the current study are available from the corresponding author on reasonable request.

## Supplementary Materials

The following supporting information can be downloaded at: https://www.mdpi.com/article/10.3390/gels10040214/s1, Figure S1: DSC curves of neat BW (a) and neat GMS (b); Figure S2: Crystallization curves of BW-based (a) and GMS-based (c) oleogels. Melting curves of BW-based (b) and GMS-based (d) oleogels.
