# Supplementary material for: A Comparative Study between Beeswax and Glycerol Monostearate for Food-Grade Oleogels
Source: Gels. 2024 Mar 22;10(4):214. doi: 10.3390/gels10040214 (PMC11049244; doi:10.3390/gels10040214)
Supplement: Supplementary file 1 [file gels-10-00214-s001.zip › gels-2926139-supplementary.pdf]

A Comparative Study between Beeswax and Glycerol Monostearate for the Food-Grade Oleogels

Francesca Malvano[1] Donatella Albanese[1]*, Luciano Cinquanta[2], Liparoti Sara[1], Francesco Marra[1]

Supplementary Materials

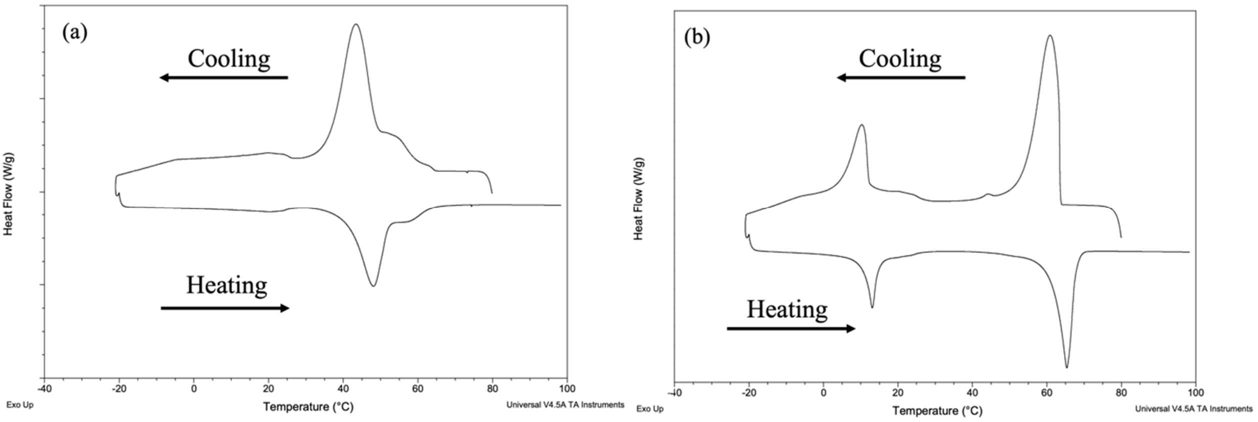

Figure S1. DSC curves of neat BW (a) and neat GMS (b)

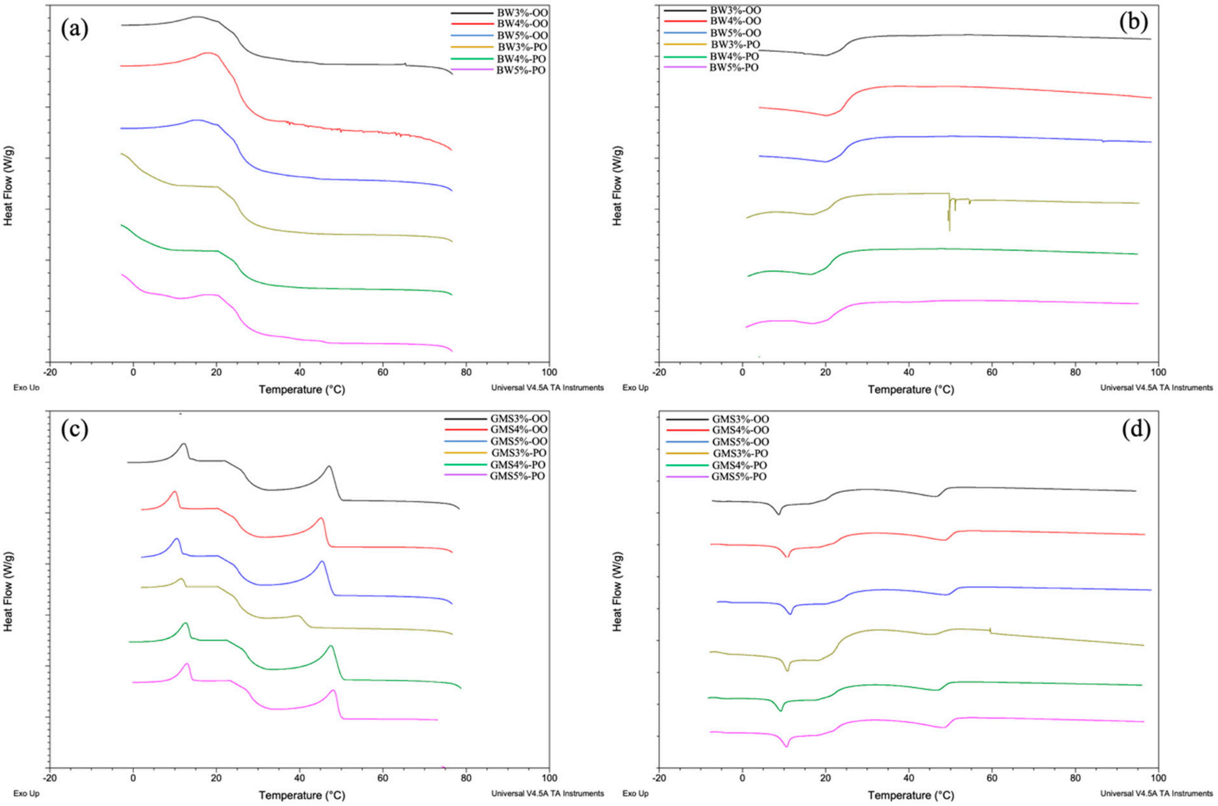

Figure S2. Crystallization curves of BW-based (a) and GMS-based (c) oleogels. Melting curves of BW-based (b) and GMS-based (d) oleogels
